# Long-term neurodevelopment in preterm neonates with necrotizing enterocolitis: systematic review and meta-analysis

**DOI:** 10.3389/fnins.2026.1794548

**Published:** 2026-05-11

**Authors:** Katrien Vandenberghe, Liselotte Van Loo, Thomas Vandendriessche, Eline Vancoppenolle, Katrien Jansen, Maissa Rayyan, Anneleen Dereymaeker

**Affiliations:** 1 Department of Development and Regeneration, KU Leuven, Leuven, Belgium; 2 Department of Neonatology, University Hospitals Leuven, Leuven, Belgium; 3 KU Leuven Libraries – 2Bergen, Leuven, Belgium; 4 Department of Pediatric Neurology, University Hospitals Leuven, Leuven, Belgium

**Keywords:** necrotizing enterocolitis, neonatal intensive care unit, neurodevelopmental outcome, very low birth weight, very preterm

## Abstract

**Introduction:**

Necrotizing enterocolitis (NEC) is a common complication in preterm infants and is associated with significant mortality and long-term morbidity, including gastrointestinal sequelae, brain injury, and developmental delays. This systematic review and meta-analysis examines long-term neurodevelopmental outcomes in infants born at less than 34 weeks’ gestation who survive NEC and identifies specific developmental domains most vulnerable to neurodevelopmental impairment.

**Methods:**

The systematic review was performed according to the PRISMA guidelines. We systematically searched Pubmed (including MEDLINE), Embase and Web of Science for relevant articles. Studies were graded for quality using the GRADE system and bias was assessed using the ROBINS-E Risk of Bias tool. We performed gestational-age stratified subgroup analyses (22–28 weeks versus 29–34 weeks) and evaluated the risk of impairment in different neurodevelopmental domains.

**Results:**

Survivors of NEC are at increased risk of neurodevelopmental impairment (RR 1.42, 95% CI 1.32–1.53). Several neurodevelopmental domains are negatively impacted, such as motor skills (RR 2.08, 95% CI 1.86–2.32), cognition (RR 1.75, 95% CI 1.57–1.96), vision (RR 4.36, 95% CI 2.91–6.55), hearing (RR 4.09, 95% CI 2.91–5.77) and cerebral palsy (RR 2.48, 95% CI 2.15–2.86). The risk of epilepsy and behavioral problems does not differ between NEC survivors and age-matched controls. This increased risk of impairment after NEC persists after stratification for gestational age and extends into school-age.

**Conclusion:**

NEC Survivors face an elevated risk of neurodevelopmental impairment, irrespective of gestational age, with deficits spanning multiple developmental domains. These findings highlight the need for targeted, long-term follow-up to enable timely detection and individualized interventions for developmental delays throughout childhood.

**Systematic review registration:**

http://www.crd.york.ac.uk/PROSPERO, identifier CRD42022322564.

## 1 Introduction

Necrotizing enterocolitis (NEC) is a severe, life-threatening gastro-intestinal disorder that affects approximately 6%–10% of preterm neonates. It is characterized by intestinal inflammation and can rapidly progress to ischemia, necrosis, and perforation (Rich and Dolgin, 2017; Imren et al., 2022; Jones and Hall, 2020). Clinical signs of NEC are often nonspecific and include abdominal distension, feeding intolerance and overt or occult fecal bleeding (Nino et al., 2016). The severity of NEC is classified using Bell’s staging (Bell et al., 1978; Walsh and Kliegman, 1986): Stage I is associated with nonspecific clinical and radiographic findings, Stage II (confirmed NEC) is marked by pneumatosis intestinalis or portal venous gas, and stage III (advanced NEC) is characterized by pneumoperitoneum. The pathogenesis of NEC involves increased intestinal reactivity in the preterm infants due to altered inflammatory dynamics (e.g., Vascular Endothelial Growth Factor (VEGF)-mediated microvascular regulation and increased Toll-like receptor-4 (TLR4)-signaling) (Zhang et al., 2025), which increases the susceptibility of the preterm bowel to tissue injury and impaired perfusion in response to microbial colonization (Nino et al., 2016).

Extremely Low Birth Weight infants (ELBW, birth weight <1,000 grams) are at highest risk (Wang et al., 2024; Stoll et al., 2015; Sharma and Hudak, 2013), with an incidence up to10% in ELBW infants (Samuels et al., 2017). Modifiable risk factors that reduce NEC risk include the administration of antenatal steroids, early enteral feeding, human milk feeding and standardized feeding protocols (Quigley et al., 2019; Jasani and Patole, 2017; Patole and de Klerk, 2005).

Management approaches vary according to disease severity, ranging from supportive and conservative measures—such as stopping enteral feeding and administering antimicrobial therapy—to surgical intervention. Despite these measures, NEC outcome carries poor prognosis, with mortality rates (Imren et al., 2022; Fitzgibbons et al., 2009) ranging from 20% to 50% depending on Bell’s staging (Stoll et al., 2015; Jiang et al., 2020), significant gastro-intestinal morbidity including intestinal strictures (12%–35%) and short bowel syndrome (20%–35%) (Heida et al., 2016; Amin et al., 2013; Han et al., 2020). Importantly, the sequelae extend beyond the gastrointestinal tract: NEC survivors face an increased risk of brain injury and subsequent neurodevelopmental impairment (NDI), with a prevalence of approximately 40%, compared to 20% in non-NEC controls (Butler et al., 2024). Specific outcomes include higher rates of cerebral palsy (CP), visual and hearing impairment. A recent systematic review by Wang et al. highlights the link between gut dysfunction and brain pathology, reporting that NDI prevalence at 1 year correlates with NEC severity, and that NEC increases the risk of brain injury [severe intraventricular hemorrhage (IVH) and periventricular leukomalacia (PVL)]. Earlier studies have largely focused on short-term neurodevelopmental outcomes following NEC, often with limited adjustment for confounding variables such as birth weight, gestational age, antenatal corticosteroid exposure, and infections.

Further research is needed to elucidate the gut–brain connection and to investigate long-term neurodevelopmental outcomes, which may help identify the specific domains most affected by NEC. Early recognition of at-risk children is essential for optimal resource allocation and timely implementation of targeted interventions.

The objective of this systematic review is to determine the overall incidence of neurodevelopmental impairment (NDI) among NEC survivors born at less than 34 weeks’ gestation, compared with age-matched controls, and to perform a subgroup analysis stratified by gestational age (22–28 weeks versus 29–34 weeks). The secondary aim is to identify specific NDI subtypes (motor, cognition, hearing, vision, learning/language and behavior), neurological complications (cerebral palsy, epilepsy) and NDI until school age in survivors of NEC.

## 2 Methods

### 2.1 Design

This study reports according to the Preferred Reporting Items for Systematic Reviews and Meta-Analyses (PRISMA) guidelines (Page et al., 2021). The study protocol was prospectively registered in the International Prospective Register for Systematic Reviews (PROSPERO, CRD42022322564, http://www.crd.york.ac.uk/PROSPERO).

### 2.2 Eligibility

Original studies on human subjects reporting on neurodevelopmental outcomes (including motor function, cognition, cerebral palsy, epilepsy, hearing or vision impairment, learning/language difficulties and behavioral disorders) after NEC (Bell’s stage ≥ IIa) in infants with GA < 34 weeks were eligible for inclusion. Outcomes should be reported either in infancy (<1 year) or at toddler (1–2 years), preschool (3–5 years), or school age (6–12 years). Studies not available in English were excluded. Studies published before the year 2000 were excluded from analysis due to the major evolution in neonatal intensive care in the last decades (Blakely et al., 2021).

### 2.3 Search strategy and data extraction

The databases PubMed (via NCBI, including MEDLINE - coverage from 2000 to date searched), Embase (via Embase.com – 2000 to date searched) and Web of Science (via webofscience.com; SCI-EXPANDED – 2000 to date searched, SSCI – 2000 to date searched, AHCI – 2000 to date searched, CPCI-S – 2000 to date searched, CPCI-SSH – 2000 to date searched, BKCI-S – 2005 to date searched, BKCI-SSH – 2005 to date searched, ESCI – 2018 to date searched), were comprehensively searched on March 21st, 2022 and the search was updated on December 6th, 2023. The full search strategy for PubMed is included in Supplement I. The deduplicated records were imported in Rayyan (Ouzzani et al., 2016) for title/abstract and full text screening. The screening was done by 3 independent reviewers (KV, LVL, ADR). Any disagreements were resolved by a third independent reviewer (MR). Data was extracted from the included manuscripts by one reviewer (KV). If insufficient data was available from the manuscript and supplementary data, authors were contacted for more information.

### 2.4 Data analysis

Data was extracted from eligible studies by one independent reviewer and included: study setting (country, year of publication, study period), study design, inclusion criteria and development assessment scales used. The following demographic data were extracted: sample size, participant demographics (birth weight, gestational age, gender), length of stay, duration of parenteral nutrition, duration of ventilation and major co-morbidities [infection, IVH, PVL, retinopathy of prematurity (ROP), bronchopulmonary dysplasia (BPD)]. Primary outcomes were overall NDI, NDI according to gestational age group. Secondary outcome was impairment in specific neurodevelopmental subdomains (cognitive, motor, hearing, vision, learning/language, behavior) and the risk of neurological complications (cerebral palsy, epilepsy).

### 2.5 Quality of evidence and risk of bias assessment

The quality of studies was independently rated by two reviewers (KV, LVL) using the GRADE system. The GRADE system assesses four items that decrease the quality of evidence (imprecision, inconsistency, indirectness and publication bias) and three items increasing study quality (large effect, dose response and confounding factors), consequently categorizing included studies into high, moderate, low or very low grade of evidence. Risk of bias was assessed for each study by two independent reviewers (KV, LVL) using the ROBINS-E tool (Higgins et al., 2024), evaluating seven domains of possible bias and resulting in a low risk, some concerns, high risk, very high risk rating. Any disagreements on grading quality or risk of bias were resolved by a third independent reviewer (ADR).

### 2.6 Statistical analysis

The relative risk of overall NDI as well as subdomain impairments between NEC survivors and age-matched controls was calculated using Cochrane’s Review Manager, The Cochrane collaboration (The Cochrane Collaboration Aarco, 2024) and graphically presented in a forest plot. Secondly, a gestational-age stratified subgroup analysis (22–28 vs. 29–34 weeks GA) was performed based on the initial conclusion criteria of the studies. Results were expressed as relative risks (RR) or Odds Ratio’s (OR). *p*-value’s <0.05 were considered statistically significant. The Chi-Square Test was performed to test heterogeneity between studies. If the included studies were sufficiently homogenous in terms of design, setting, population and outcome measures, the ORs for neurodevelopmental disorders were pooled and a fixed-effect meta-analysis was performed.

## 3 Results

### 3.1 Study selection

**Figure 1:**
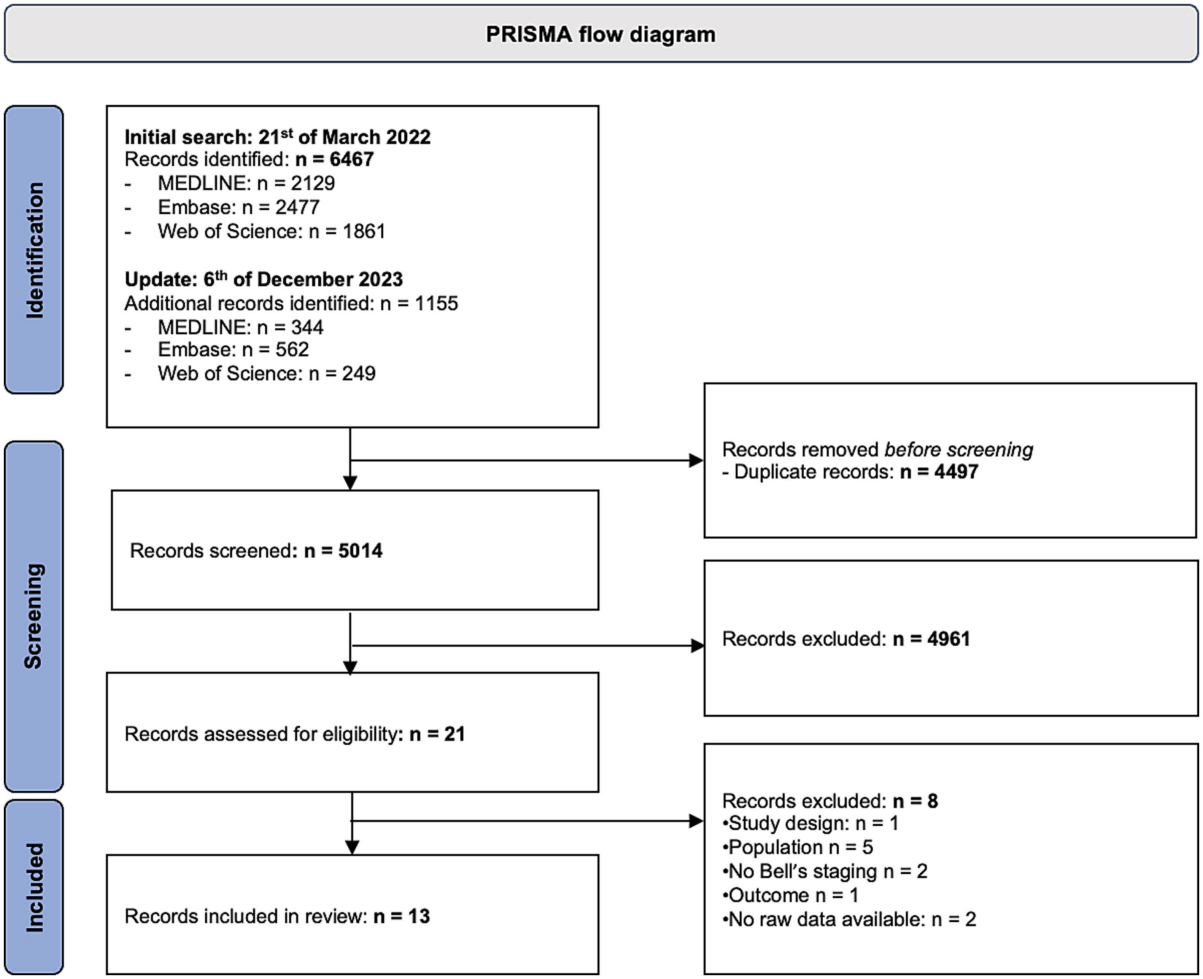
PRISMA flow diagram of included studies.

The PRISMA flow diagram is presented in Figure 1. Initial search was performed on March 21st, 2022 and yielded 6,467 records. The search was updated on December 6th, 2023 which identified 1,155 additional publications. After screening and eligibility assessment, 13 individual studies were included in the final review.

### 3.2 Study characteristics

Table 1 provides an overview of the main study characteristics. This review included thirteen studies (Imren et al., 2022; Han et al., 2020; Blakely et al., 2021; Berry et al., 2019; Chen et al., 2021; Shin et al., 2021; Dilli et al., 2012; Martin et al., 2010; Shah et al., 2012; Vaidya et al., 2022; Wadhawan et al., 2014; Zozaya et al., 2021; Fullerton et al., 2017): five retrospective cohort studies, three prospective case–control studies, four retrospective case–control studies and one randomized controlled trial. The start of the recruitment period varied from 1998 to 2017. Patients were followed until a median age of 24 corrected months. Only 2 studies reported outcomes beyond toddlerhood, up until 15 years of age (Han et al., 2020; Vaidya et al., 2022). All but one study (Han et al., 2020) used a validated developmental assessment tool to report neurodevelopmental outcome: 10/13 studies reported outcomes using the Bayley Scales of Infant and Toddler Development (BSID) (Balasundaram and Avulakunta, 2022).

**Table 1:** Characteristics of included studies.

Author	Country	Design	Inclusion criteria	Inclusion period	Corrected age at follow-up (months)	Developmental test
Berry et al. (2019)	New Zealand	Cohort (retrospective)	23^0^–24^6^	2002–2017	24–36	BSID- II/III or GMDS
Blakely et al. (2021)	USA	Multicenter randomized controlled trial (prospective)	<1,000 g, <8w, surgical	2010–2017	18–22	BSID-III
Chen et al. (2021)	China	Case–control (prospective)	29–32w, Bell’s ≥ II	2017–2018	12–18	GDS
Dilli et al. (2012)	Turkey	Case–control (retrospective)	29–32w, Bell’s ≥ II	2007–2009	18–24	BSID-II GMFCS
Fullerton et al. (2017)	USA & Canada	Case–control (retrospective)	22-27w	1999–2012	18–24	BSID-II/III
Han et al. (2020)	USA	Cohort (retrospective)	Severe surgical NEC (<30 cm bowel left)	2009–2018	48–120	No scale used
Imren et al. (2022)	the Netherlands	Cohort (retrospective)	Bell’s ≥ II	2008–2020	24	BSID-III
Martin et al. (2010)	USA and Germany	Case–control (prospective)	23–27w	2002–2004	24	BSID-II
Shah et al. (2012)	USA	Cohort (retrospective)	Bell ≥2A ≤ 1,000 g	1998–2009	18–22	BSID-II
Shin et al. (2021)	Korea	Cohort (retrospective)	<32w, surgical NEC	2006–2018	24 & 36	BSID-III (24 months), K-ASQ or K-DST (36 months)
Vaidya et al. (2022)	USA	Case–control (prospective)	23–27w	2002–2004	120 & 180	DAS-II FSIQ WASI-II LPA GMFCS
Wadhawan et al. (2014)	USA	Case–control (retrospective)	≤1,000 g, alive >12 h	2000–2005	18–22	BSID-II
Zozaya et al. (2021)	Canada	Case–control (retrospective)	22–28^6^w	2010–2011	18–24	BSID-III

USA, United States of America; BSID, Bayley scales of infant & toddler development (version II or III); GMDS, Griffiths developmental scales; GDS, Gesell developmental schedules; FSIQ, full-scale IQ; WASI-II, Wechsler abbreviated scale of intelligence-II; K-DST, Korean developmental screening test; DAS-II, school-age differential ability scales-II; LPA, latent profile analysis; GMFCS, gross motor function classification system.

Participant characteristics are represented in Table 2. Eleven studies reported on neonates with GA 22–28 weeks and only two studies reported on preterm neonates with GA between 29 and 34 weeks (Chen et al., 2021; Dilli et al., 2012). The weighted mean GA of NEC survivors across studies was 25.6 weeks (SD 2.01).

**Table 2:** Participant characteristics.

Author name	BW (grams) mean ± SD or median (range)	GA (weeks) mean ± SD or median(range)	Gender (%male)	LOS (days)	PN (days) mean ± SD	Ventilation (days)	Infection (%)	IVH (%)	PVL (%)	ROP (%)	BPD (%)
Berry et al. (2019) NEC, perforation (*n* = 8) NEC, no perforation(*n* = 33) Controls (*n* = 133)	675 (585⁠–⁠800) 656 (465⁠–⁠955) 669 (430⁠–⁠970)	23⁠^0^⁠–⁠24⁠^6^	88 47 55	125 (34⁠–⁠186) 85 (12⁠–⁠348) 85 (12⁠–⁠348)	NS	NS	NS	0 12 10	NS	NS	NS
Blakely et al. (2021) NEC, Laparotomy (*n* = 42) NEC, Drainage (*n* = 109)	721 711	25.1 24.9	55 61	80 99	54 65	31 32	26 33	2.6 5.9	NS	NS	NS
Chen et al. (2021) NEC (*n* = 11) Controls (n = 17)	1,255 1,324	29.1 29.4	100 26	NS	NS	NS	NS	0 0	0 0	27 12	27 41
Dilli et al. (2012) NEC (*n* = 20) Controls (*n* = 40)	1,065 (940⁠–⁠1,272) 1,240 (1000⁠–⁠1,367)	28.5 (27⁠–⁠30) 28.5 (28⁠–⁠30.7)	45 60	56 (35⁠–⁠69) 41 (28⁠–⁠47)	NS	NS	NS	NS	NS	NS	NS
Fullerton et al. (2017) Surgical NEC (*n* = 449) Medical NEC (*n* = 417) Controls (*n* = 9,063)	752 ± 172 779 ± 162 807 ± 169	25 ± 2 26 ± 2 26 ± 2	58 53 49	124 100 87	NS	NS	55 41 27	NS	NS	32 21 16	58 52 46
Han et al. (2020) (*n* = 41)	930 (IQR 740⁠–⁠1,246)	28 (IQR 26⁠–⁠31)	61	NS	1,298 (IQR 778⁠–⁠3,183)	NS	NS	25 (any IVH)	NS	34	39
Imren et al. (2022) (*n* = 200)	855 (IQR 700⁠–⁠1,058)	26.4 (IQR 25.3⁠–⁠28.11)	58	NS	NS	NS	NS	NS	NS	NS	NS
Martin et al. (2010) Medical NEC (*n* = 59) Surgical NEC (*n* = 42) Controls (*n* = 1,054)	NS	23⁠–⁠27	NS	NS	NS	NS	34 33 23	NS	NS	NS	NS
Shah et al. (2012) NEC (*n* = 208) Medical (*n* = 87) Surgical (*n* = 121) Controls (*n* = 1,459)	759 ± 145 69 ± 140 753 ± 148 783 ± 144	25.9 ± 2 26.1 ± 1.8 25.7 ± 2.1 26.2 ± 2	51 52 51 46	103 ± 21 94 ± 22 112 ± 15 79 ± 26	44 ± 33 38 ± 23 49 ± 38 19 ± 15	26 ± 27 20 ± 24 30 ± 28 15 ± 23	39 32 45 22	11 17 6 6	6 7 6 3	51 62 44 55	26 28 26 35
Shin et al. (2021) Surgical NEC (*n* = 60)	710 (IQR 606⁠–⁠980)	26.1 (IQR 24.3⁠–⁠27.5)	38	NS	NS	NS	58	18	22	NS	62
Vaidya et al. (2022) Medical NEC (*n* = 138) Surgical NEC (*n* = 33) Controls (n = 689)	Z-score −0.48 ± 1.33 −0.81 ± 1.27 −0.75 ± 1.34	23⁠–⁠27	50 70 50	NS	NS	NS	NS	NS	NS	19 27 11	46 36 41
Wadhawan et al. (2014) Surgical NEC (*n* = 472) Controls (*n* = 8,184)	736 ± 142 765 ± 147	25.6 ± 1.9 25.9 ± 2.1	58 49	101 ± 78 81 ± 53	57 ± 36 27 ± 19	NS	63 36	23 18	11 5	NS	67 48
Zozaya et al. (2021) NEC, perforation (*n* = 61) NEC, no perforation (*n* = 115) Controls (*n* = 1804)	862 ± 229 871 ± 238 909 ± 241	25.4 ± 1.7 25.8 ± 1.4 26.1 ± 1.5	72 61 52	NS	NS	NS	NS	20 15 15	NS	23 30 14	63 53 45

NEC, necrotizing enterocolitis; IQR, interquartile range; SD, standard deviation; BW, birth weight; GA, gestational age; LOS, length of hospital stay; PN, parenteral nutrition; IVH, intraventricular hemorrhage grade 3–4; PVL, periventricular leukomalacia; ROP, retinopathy of prematurity; BPD, bronchopulmonary dysplasia; NS, not specified.

### 3.3 Results of included studies

The neurodevelopmental outcomes of individual studies are shown in Table 3. 9/13 studies reported the incidence of overall NDI. The study by Shah et al. (31) only reported ORs without absolute values and the data of that study were therefore not included in further statistical analyses. A total of 2,518 NEC survivors and 22.443 controls were initially included, and individual outcome data was available on 2,302 NEC survivors and 21.220 controls for inclusion in the meta-analysis. Multiple studies additionally reported outcomes on neonates who experienced spontaneous intestinal perforation (SIP), they were omitted from further analysis as it is beyond the scope of this review.

**Table 3:** Neurodevelopmental outcomes after NEC.

Corrected age at follow up	N assessed	Over allNDI (%)	Type of NDI/comorbidity (%)							
			Motor	Cognitive	Hearing	Vision	Learning/language	Behavior	Cerebral palsy	Epilepsy
Toddler age (1–2 yr)										
12–18 months (Chen et al., 2021) NEC Controls	11 17	82 24	NS	NS	NS	NS	NS	NS	NS	NS
Blakely et al. (2021) NEC	94	31	NS	NS	NS	NS	NS	NS	NS	NS
18–24 months (Shin et al., 2021) Surgical NEC	31	52	48	39	NS	NS	39	NS	NS	NS
Dilli et al. (2012) NEC Controls	20 40	55 30	NS	NS	NS	NS	NS	NS	1010	NS
Fullerton et al. (2017) NEC Controls	866 9,063	NS	18 8	14 8	3.1 1.4	2.1 0.6	NS	NS	15 7	1.7 1.1
Shah et al. (2012) NEC Controls	52 785	OR 2.59 (95% CI 1.44⁠–⁠4.66)	OR 2.64 (95% CI 1.18⁠–⁠5.91)	NS	NS	NS	NS	NS	NS	NS
Wadhawan et al. (2014) Surgical NEC Controls	472 8,184	57 36	46 20	51 30	8 0.6	5 0.6	NS	NS	23 6	NS
Zozaya et al. (2021) NEC Controls	176 1843	39 37	NS	NS	NS	NS	NS	NS	NS	NS
24 months (Shin et al., 2021) Surgical NEC	42	52	NS	NS	12	7.1	NS	NS	17	NS
Imren et al. (2022)	72	NS	26	25	NS	NS	NS	NS	4	NS
Martin et al. (2010) Medical NEC Surgical NEC Controls	28 39 809	NS	29 48 27	23 39 22	NS	NS	NS	NS	15 14 13	NS
24–36 months (Berry et al., 2019) NEC Controls	19 35	47 46	11 2.9	11 11	0 11	5 6	8 6	NS	NS	NS
Preschool age (3–5 yr)										
36 months: (Shin et al., 2021) Surgical NEC	27	NS	48	56	NS	NS	52	44	NS	NS
School age (6–12 yr)										
4–10 years (Han et al., 2020)	28	NS	18	NS	21	7	NS	NS	14	29
10 years (Vaidya et al., 2022) NEC Controls	271 689	NS	NS	NS	NS	NS	NS	16 17	9 8	7 8
15 years (Vaidya et al., 2022) NEC Controls	133 540	NS	NS	NS	NS	NS	NS	7 6	NS	8 7

NEC, necrotizing enterocolitis; NS, not specified.

Most (8/13) studies reported on motor dysfunction or CP (Imren et al., 2022; Han et al., 2020; Berry et al., 2019; Shin et al., 2021; Dilli et al., 2012; Martin et al., 2010; Shah et al., 2012; Wadhawan et al., 2014; Fullerton et al., 2017). Five studies reported on the prevalence of cognitive impairment (Berry et al., 2019; Shin et al., 2021; Martin et al., 2010; Wadhawan et al., 2014; Fullerton et al., 2017). Two studies reported language difficulties (Berry et al., 2019; Shin et al., 2021), three studies reported epilepsy rates (Han et al., 2020; Vaidya et al., 2022; Fullerton et al., 2017), five studies reported on the prevalence of hearing impairment and blindness (Han et al., 2020; Berry et al., 2019; Shin et al., 2021; Wadhawan et al., 2014; Fullerton et al., 2017). One study (Shin et al., 2021) explored social functioning and another study reported on the occurrence of Attention Deficit Hyperactivity Disorder (ADHD) in infants with NEC (Vaidya et al., 2022).

### 3.4 Meta-analysis

**Figure 2:**
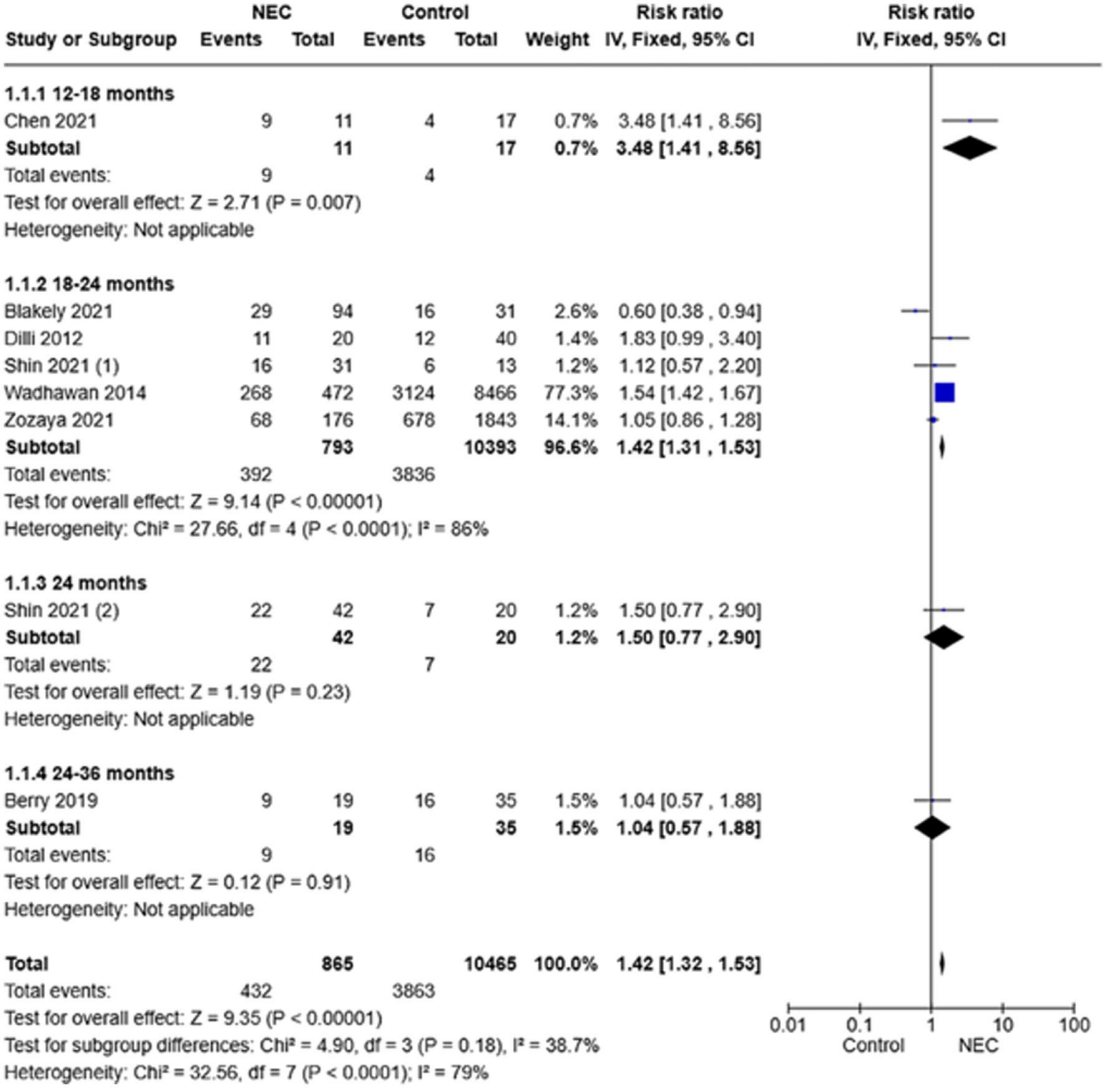
Forest plot of fixed-effect analysis comparing the overall risk of neurodevelopmental impairment in NEC survivors versus controls.

The results of the twelve studies included in this systematic review were combined into a meta-analysis comparing the overall incidence of NDI in NEC survivors and age-matched controls. Overall, NEC survivors had a significantly higher risk of NDI (RR 1.42, 95% CI 1.32–1.53) (Figure 2). Overall heterogeneity of included studies was high (Chi^2^ = 32.56, *p* < 0.0001, I^2^ = 79%).

**Figure 3:**
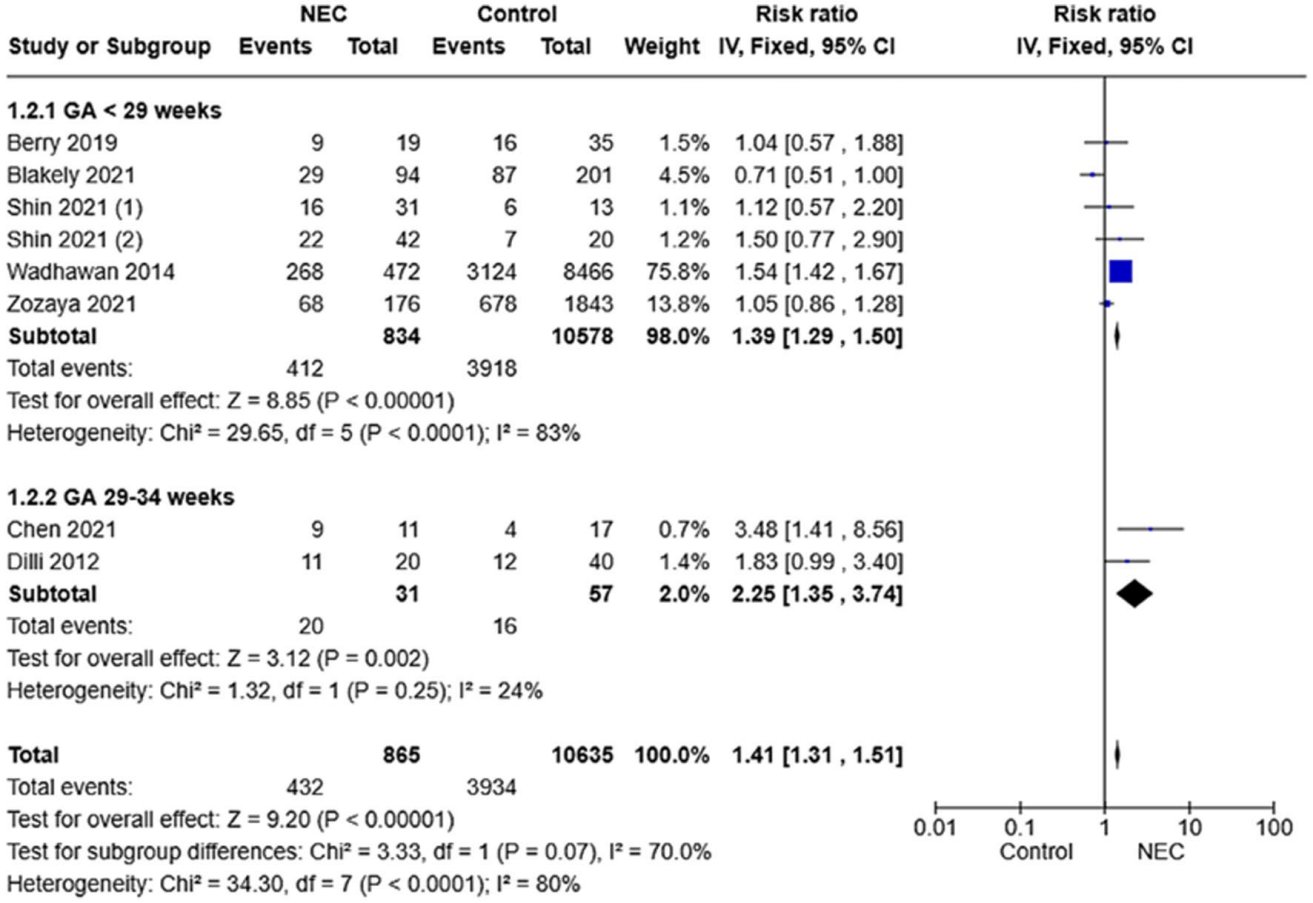
Forest plot of fixed-effect analysis comparing the risk of neurodevelopmental impairment divided by gestational-age group (<29 weeks versus 29–34 weeks) in NEC survivors versus controls.

Subgroup analysis showed that both patients with GA 22–28 weeks (RR 1.39, 95% CI 1.29–1.50) and preterm neonates with GA 29–32 weeks (RR 2.25, 95% CI 1.35–3.74) had increased risk of NDI after NEC. No studies reported outcomes on patients with GA 33–34 weeks. Forest plots are shown in Figure 3.

**Figure 4:**
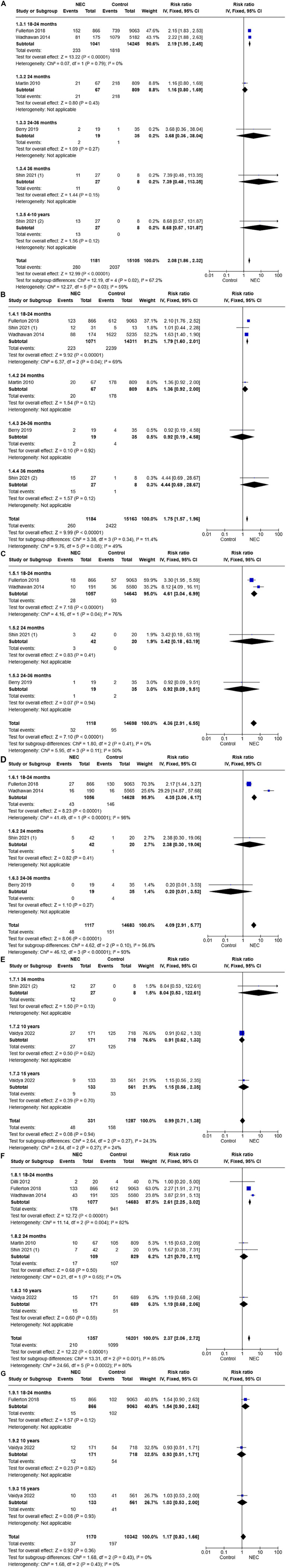
Forest plot of fixed-effect analysis comparing the risk of impairment in specific neurodevelopmental domains and neurological complications in NEC survivors versus controls: **(A)** Motor impairment, **(B)** cognitive impairment, **(C)** visual impairment, **(D)** hearing impairment, **(E)** behavioural difficulties, **(F)** cerebral palsy, **(G)** epilepsy.

**Figure 5:**
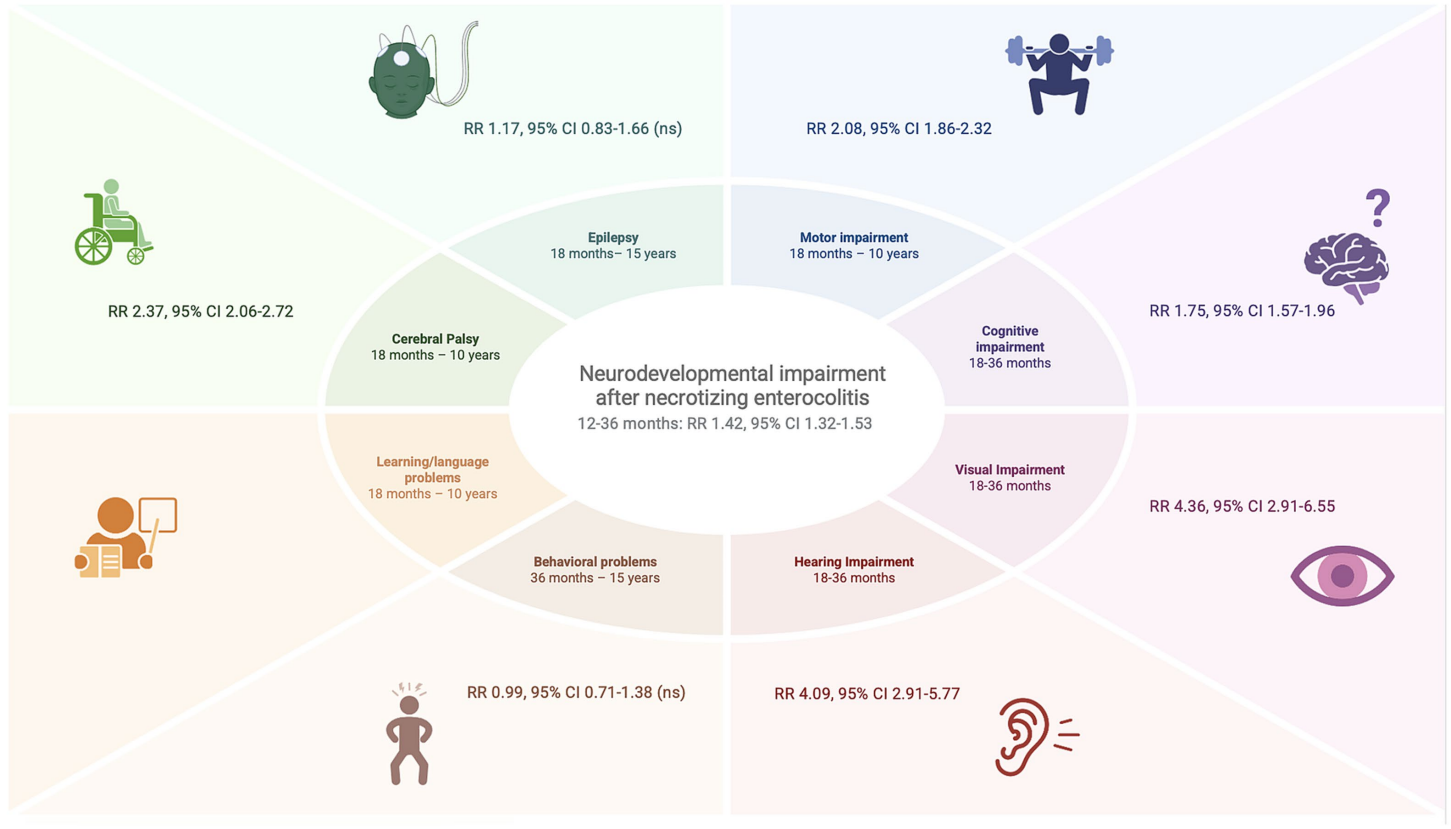
Visual representation of the results of the meta-analysis. RR, risk ratio.

When subdividing the results in developmental domains, motor (RR 2.08, 95% CI 1.86–2.32), cognitive (RR 1.75, 95% CI 1.57–1.96), visual (RR 4.36, 95% CI 2.91–6.55) and hearing (RR 4.09, 95% CI 2.91–5.77) were all more prevalent in the NEC group. In addition, NEC survivors had significantly higher risk of developing CP compared to age-matched controls (RR 2.37, 95% CI 2.06–2.72). No significant difference was found in the prevalence of epilepsy (RR 1.17, 95% CI 0.83–1.66) and behavioral disorders (RR 0.99, 95% CI 0.71–1.38). For learning/language difficulties, no meta-analysis was performed as only one study included a control group. Forest plots are presented in Figures 4A–G and a visual overview of the results is presented in Figure 5.

### 3.5 Quality of evidence and risk of bias

GRADE of evidence was deemed high in one study (Blakely et al., 2021), moderate in four studies (Chen et al., 2021; Martin et al., 2010; Vaidya et al., 2022; Zozaya et al., 2021), low in seven studies (Imren et al., 2022; Berry et al., 2019; Shin et al., 2021; Dilli et al., 2012; Shah et al., 2012; Wadhawan et al., 2014; Fullerton et al., 2017) and very low in one (Han et al., 2020). The overall risk of bias was evaluated as low in one study (Chen et al., 2021), some concerns in six studies (Blakely et al., 2021; Shin et al., 2021; Martin et al., 2010; Shah et al., 2012; Vaidya et al., 2022; Zozaya et al., 2021), high in five studies (Imren et al., 2022; Berry et al., 2019; Dilli et al., 2012; Wadhawan et al., 2014; Fullerton et al., 2017), and very high in one study (Han et al., 2020). A detailed overview of the GRADE and ROBINS-E assessments is provided in Supplement II.

## 4 Discussion

This systematic review with meta-analysis reports on neurodevelopmental outcomes of 2,518 NEC survivors born before 34 weeks GA, compared to 22.443 age-matched controls. It reviews overall incidence of NDI as well as the incidence of deficits in neurodevelopmental subdomains (motor, cognition, vision, hearing, learning/language and behavior) and neurological complications (cerebral palsy, epilepsy). Lastly, we performed a subgroup analysis of NEC survivors born between 22 and 28 versus 29–34 weeks GA.

Our findings align with two previously published reviews: Matei et al. found a prevalence of NDI of around 40% after surviving NEC, an increased risk of CP as well as an increased prevalence of brain injuries (IVH and PVL) in NEC survivors (Matei et al., 2020). Additionally, they documented an association of NDI prevalence and NEC severity. However, this study included children across all gestational ages (including term infants), exhibited substantial statistical heterogeneity, and lacked stratification for key confounders such as gestational age, birth weight, infection rates, and antenatal steroid exposure. The systematic review by Wang et al. reported a higher incidence of cerebral palsy—although this association lost significance after adjustment for confounders—as well as vision and hearing impairments, with a greater risk observed in surgical NEC compared to medical NEC. Follow-up duration was limited to 2 years of age (Wang et al., 2024).

The results from this review enforce current knowledge that NEC is not only a short-term gastro-intestinal complication in the neonatal period but has detrimental neurodevelopmental consequences continuing into school age. On one hand, direct brain injury during the acute illness (IVH, PVL); on the other hand, dysmaturational processes affect further brain development (Inder et al., 2023). Possible mechanism for this gut-brain connection are intestinal microbiome dysregulation and altered cytokine expression (Manohar et al., 2023; Lu et al., 2023), combined with immaturity of the blood–brain barrier and increased vulnerability of oligodendrocytes in the preterm brain to the inflammatory cascade (Boccazzi et al., 2021; Bennet et al., 2018). In addition, accompanying hemodynamic instability including hypotension and acidosis, perioperative complications and prolonged ventilation might contribute to this neurodevelopmental risk (Selvanathan et al., 2023). Lastly, prolonged parenteral feeding and omission of enteral feeds could disturb normal growth and development (Itriago et al., 2023; Premkumar et al., 2022).

The increased risk of impairment in specific neurodevelopmental domains persisting into school age shows the need for tailored follow-up including validated assessment tools beyond toddlerhood. Included manuscripts used the BSID most often, although it only reports on motor and cognitive function and the BSID scales tend to underestimate the extent of gross motor deficit. For future research, other assessments such as early executive function (EF) can be of value. Early EF assessment is feasible from approximately 2 years of age (e.g., working memory, inhibitory control, and cognitive flexibility (Van den Brande et al., 2024)), and represents a particularly informative target in the search for early markers of atypical development. Executive function reflects the integrity of early brain networks and begins to emerge well before formal diagnoses of other neurodevelopmental conditions can be established. Because EF deficits occur across a wide spectrum of conditions—including autism spectrum disorder, attention-deficit/hyperactivity disorder, and learning disabilities, all of which are more prevalent in preterm populations—EF functions as a sensitive early indicator of neurodevelopmental vulnerability. Furthermore, EF performance robustly predicts long-term behavioral, social, and academic outcomes. Importantly, EF is also modifiable through early intervention, strengthening its relevance as an early, actionable marker of developmental risk in children born preterm. At school the Wechsler Intelligence Scale for Children for cognition, the Movement Assessment Battery for Children–2 for motor function and the Behavior Rating Inventory of Executive Function (BRIEF) (Dodzik, 2017) might provide continued insight into the full neurodevelopmental profile of NEC survivors.

In this analysis, the relative risk of NDI was even more pronounced in patients with higher GA compared to extreme preterm neonates 22–28 weeks GA.

These findings may be influenced by the small number of studies and by differences in their inclusion criteria—specifically, the focus on surgical NEC in one study compared with the broader populations in others. A higher proportion of infants requiring abdominal surgery may have increased neurodevelopmental risk likely representing a more severely ill population. Moreover, additional comorbidities (e.g., growth restriction) could also have contributed to worser outcomes. Nevertheless, these results highlight the importance of close neurodevelopmental follow-up for all NEC survivors, regardless of gestational age, and underscore the need for particular attention to older NEC survivors during long-term developmental surveillance.

### 4.1 Limitations

The included studies were observational in design, predominantly retrospective, and did not consistently include an age-matched control group, all of which represent potential sources of bias. Additionally, substantial heterogeneity was observed across studies, primarily attributable to differences in inclusion criteria and to variability in the definition of neurodevelopmental impairment (NDI) and the use of multiple developmental assessment scales. As not all studies reported outcomes across neurodevelopmental subdomains, the conclusions regarding domain-specific impairments are based on limited data.

These findings should be interpreted with caution, as potential confounding factors were not uniformly accounted for. Neurodevelopmental impairment in preterm infants is multifactorial and is commonly associated with factors such as prolonged invasive ventilation, systemic inflammation, acquired brain injury, nutritional challenges, exposure to analgosedative medications, and extended hospital stay (Rogers and Hintz, 2016; Ream and Lehwald, 2018). Although many studies adjusted for selected confounding factors, we were unable to comprehensively account for the full spectrum of comorbidities associated with extreme prematurity. In addition, the included studies span the period from 2000 to 2023, during which substantial advances in neonatal care have occurred. As only one additional eligible study has been published since the original search, the meta-analysis was not updated (Culbreath et al., 2024).

### 4.2 Strengths

Key strengths of this study include a comprehensive multi-database search and a rigorous study selection process performed independently by two reviewers using strict inclusion criteria. Limiting inclusion to studies published from March 2000 onward enhanced applicability to current neonatal care. Importantly, this is the first systematic review to evaluate neurodevelopmental outcomes beyond toddlerhood, allowing assessment of more complex domains, including learning and behavioural functioning, up to 15 years of age.

## 5 Conclusion

These findings support the growing consensus that preterm infants suffering from NEC are at increased risk of NDI compared to preterm neonates without NEC and that these differences persist in school age. Specifically, the risk of motor, cognitive, hearing and visual impairment, is increased and cerebral palsy is more prevalent, highlighting the need for extended neurodevelopmental follow up to enable timely detection and intervention.

Further research is necessary to clarify causal pathways and correct for other confounders such as inflammation and infection. In addition, studies examining long term outcomes beyond toddlerhood represent a small part of the included manuscript and more research on the impact of learning/language difficulties as well as behavioral issues are necessary.

## Data Availability

The original contributions presented in the study are included in the article/Supplementary material, further inquiries can be directed to the corresponding author.

## Glossary

ADHD: Attention deficit hyperactivity disorder
ASD: Autism spectrum disorder
BPD: Bronchopulmonary dysplasia
BRIEF: Behavior rating inventory of executive function
BSID: Bayley scales of infant and toddler development
EC: Ethical committee
EF: Executive function
ELBW: Extremely low birth weight
GA: Gestational age
IVH: Intraventricular hemorrhage
NDI: Neurodevelopmental impairment
NEC: Necrotizing enterocolitis
OR: Odds Ratio
PVL: Periventricular leukomalacia
ROP: Retinopathy of prematurity
RR: Relative risk
SIP: Spontaneous intestinal perforation
SR: Systematic review
TLR4: Toll-Like receptor-4
VEGF: Vascular endothelial growth factor
