# Supplementary material for: Long-term neurodevelopment in preterm neonates with necrotizing enterocolitis: systematic review and meta-analysis
Source: Front Neurosci. 2026 May 11;20:1794548. doi: 10.3389/fnins.2026.1794548 (PMC13200335; doi:10.3389/fnins.2026.1794548)
Supplement: Supplementary file 1 [file Data_Sheet_1.DOCX]

**Supplementary Materials**

# I Search Strategy

Concept 1: necrotizing enterocolitis

"Enterocolitis, Necrotizing"[Mesh] OR “necrotizing enterocolitis”[tiab] OR “necrotising enterocolitis”[tiab] OR NEC[tiab]

Concept 2: preterms

"Infant, Newborn"[Mesh] OR “baby”[tiab] OR “babies”[tiab] OR “low birthweight”[tiab] OR “birth”[tiab] OR “born”[tiab] OR “day old”[tiab] OR “days old”[tiab] OR “gestation*”[tiab] OR “immatur*”[tiab] OR ”infant*”[tiab] OR “infancy”[tiab] OR “matur*”[tiab] OR “neonat*”[tiab] OR “neo-nata*”[tiab] OR “neo-nate*”[tiab] OR “newborn*”[tiab] OR “NICU”[tiab] OR “offspring”[tiab] OR “perinatal”[tiab] OR “peri-natal”[tiab] OR “postnatal*”[tiab] OR “post-natal*”[tiab] OR “postpartum”[tiab] OR “prematur*”[tiab] OR “pre-matur*”[tiab] OR “pre-natal”[tiab] OR “prenatal”[tiab] OR “preterm”[tiab] OR “pre-term”[tiab] OR LBW[tiab] OR VLBW[tiab] OR ELBW[tiab] OR MPT[tiab] OR VPT[tiab] OR EPT[tiab] OR ELGAN[tiab] OR ((22[tiab] OR 23[tiab] OR 24[tiab] OR 25[tiab] OR 26[tiab] OR 27[tiab] OR 28[tiab] OR 29[tiab] OR 30[tiab] OR 31[tiab] OR 32[tiab] OR 33[tiab] OR 34[tiab] ) AND (week*[tiab]))

Concept 3: outcome (long term)(no short term)

"Neurodevelopmental Disorders"[Mesh:NoExp] OR "Intellectual Disability"[Mesh:NoExp] OR ((“neurodevelopmental*”[tiab] OR “psychologic*”[tiab] OR “cognit*”[tiab] OR “intellectual*”[tiab] OR “mental”[tiab] OR “brain”[tiab] ) AND (“disorder*”[tiab] OR “disabilit*”[tiab] OR “impair*”[tiab] OR “deviat*”[tiab] OR “deficien*”[tiab] OR “retard*”[tiab] OR “outcome*”[tiab] OR “dysfunction*”[tiab] OR “trouble”[tiab] OR “difficult*”[tiab] OR “delay*”[tiab] OR “disturb*”[tiab] OR “illness”[tiab] OR “abnormal*”[tiab] OR “disease*”[tiab] OR “chang*”[tiab] OR “confus*”[tiab] OR “insufficien*”[tiab] OR “ill” [tiab] OR “aberra*”[tiab] OR “disrupt*”[tiab] OR “dysfunction*”[tiab])) OR

"Attention Deficit and Disruptive Behavior Disorders"[Mesh] OR "Problem Behavior"[Mesh] OR “disruptive behavio*”[tiab] OR ADHD[tiab] OR “attention deficit”[tiab] OR “hyperkinetic syndrome*”[tiab] OR ADDH[tiab] OR ADD[tiab] OR

((“behav*”[tiab] OR “reactive”[tiab] OR “neurobehavio*”[tiab] OR “conduct”[tiab]) AND (“disorder*”[tiab] OR “disabilit*”[tiab] OR “impair*”[tiab] OR “devia*”[tiab] OR “deficien*”[tiab] OR “retard*”[tiab] OR “outcome*”[tiab] OR “dysfunction*”[tiab] OR “trouble”[tiab] OR “difficult*”[tiab] OR “delay*”[tiab] OR “disturb*”[tiab] OR “illness” [tiab] OR “abnormal*”[tiab] OR “disease*”[tiab] OR “chang*”[tiab] OR “defect*”[tiab] OR “symptom*”[tiab] OR “ill”[tiab] OR “aberra*”[tiab] OR “crisis*”[tiab] OR “manifest*”[tiab] OR “disrupt*”[tiab] OR “dysfunction*”[tiab])) OR

"Child Development Disorders, Pervasive"[Mesh] OR ((“development*”[tiab]) AND (“disorder*”[tiab] OR “disabilit*”[tiab] OR “impair*”[tiab] OR “deviat*”[tiab] OR “deficien*”[tiab] OR “retard*”[tiab] OR “outcome*”[tiab] OR “dysfunction*”[tiab] OR “trouble”[tiab] OR “difficult*”[tiab] OR “delay*”[tiab] OR “disturb*”[tiab])) OR autis*[tiab] OR spectrum[tiab] OR ASS[tiab] OR

"Communication Disorders"[Mesh:NoExp] OR "Language Disorders"[Mesh:NoExp] OR "Language Development Disorders"[Mesh:NoExp] OR "Speech Disorders"[Mesh:NoExp] OR "Learning Disabilities"[Mesh:NoExp] OR ((“communicat*”[tiab] OR “language*”[tiab] OR “articulat*”[tiab] OR “learning”[tiab] OR “speech”[tiab] OR “speak*”[tiab]) AND (“disorder*”[tiab] OR “disabilit*”[tiab] OR “impair*”[tiab] OR “deviat*”[tiab] OR “deficien*”[tiab] OR “retard*”[tiab] OR “outcome*”[tiab] OR “dysfunction*”[tiab] OR “trouble”[tiab] OR “difficult*”[tiab] OR “delay*”[tiab] OR “unintelligible”[tiab] OR “disturb*”[tiab] OR “problem*”[tiab])) OR

"Motor Skills Disorders"[Mesh] OR "Psychomotor Disorders"[Mesh:NoExp] OR ((“Motor*”[tiab] OR “psychomotor*”[tiab] OR “coordinat*”[tiab]) AND (“disorder*”[tiab] OR “disabilit*”[tiab] OR “impair*”[tiab] OR “deviat*”[tiab] OR “deficien*”[tiab] OR “retard*”[tiab] OR “outcome*”[tiab] OR “dysfunction*”[tiab] OR “trouble”[tiab] OR “difficult*”[tiab] OR “delay*”[tiab] OR “disturb*”[tiab])) OR

"Cerebral Palsy"[Mesh] OR “cerebral pals*”[tiab] OR “diplegia*”[tiab] OR “brain pals*”[tiab] OR “central pals*”[tiab] OR “cerebral paralysis”[tiab] OR “brain paralysis”[tiab] OR “central paralysis*”[tiab] OR “cerebral paresis”[tiab] OR “brain paresis”[tiab] OR “central paresis”[tiab] OR “CP”[tiab] OR

"Epilepsy"[Mesh:NoExp] OR “epilep*”[tiab] OR “seizure*”[tiab] OR “seizing”[tiab] OR “convuls*”[tiab] OR

"Vision Disorders"[Mesh] OR “blind*”[tiab] OR ((vision[tiab] OR visual[tiab]) AND (“loss”[tiab] OR “disorder*”[tiab] OR “disabilit*”[tiab] OR “impair*”[tiab] OR “deviat*”[tiab] OR “deficien*”[tiab] OR “retard*”[tiab] OR “outcome*”[tiab] OR “dysfunction*”[tiab] OR “trouble”[tiab] OR “difficult*”[tiab] OR “delay*”[tiab] OR “disturb*”[tiab] OR “abnormal”[tiab] OR “defect*”[tiab] OR “damag*”[tiab])) OR CVI[tiab] OR

"Hearing Loss"[Mesh] OR “deaf*”[tiab] OR ((“hearing*”[tiab] OR “audit*”[tiab]) AND (“loss”[tiab] OR “disorder*”[tiab] OR “disabilit*”[tiab] OR “impair*”[tiab] OR “deviat*”[tiab] OR “deficien*”[tiab] OR “retard*”[tiab] OR “outcome*”[tiab] OR “dysfunction*”[tiab] OR “trouble”[tiab] OR “difficult*”[tiab] OR “delay*”[tiab] OR “disturb*”[tiab] OR “defect*”[tiab] OR “damage*”[tiab])) OR

"Reactive Attachment Disorder"[Mesh] OR "Affective Symptoms"[Mesh] OR ((“attachment”[tiab] OR emotional[tiab] OR “psychosocial”[tiab] OR “conduct*”[tiab] OR “affective”[tiab]) AND (“disorder*”[tiab] OR “disabilit*”[tiab] OR “impair*”[tiab] OR “deviat*”[tiab] OR “deficien*”[tiab] OR “retard*”[tiab] OR “trouble”[tiab] OR “difficult*”[tiab] OR “delay*”[tiab] OR disturb*[tiab] OR “problem*”[tiab] OR “illness*”[tiab] OR “handicap*”[tiab] OR “dysfunction*”[tiab] OR “symptom*”[tiab])) OR

"Executive Function"[Mesh] OR “executive function*”[tiab] OR “executive control*”[tiab]

- Concept 1 AND Concept 2 AND Concept 3

Time:

(("2000/01/01"[Date - Publication] : "3000"[Date - Publication]))

Update 06/12/2023:

field code ("2022/04/01"[Date - Create] : "3000"[Date - Create]);  n= 344

# II GRADE assessment and Risk of Bias

## IIa GRADE quality of evidence assessment

| Author | GRADE |
| --- | --- |
| Berry MJ (19) | Low |
| Blakely ML (20) | High |
| Chen S (21) | Moderate |
| Dilli D (22) | Low |
| Fullerton BS (23) | Low |
| Imren C (25) | Low |
| Martin CR (26) | Moderate |
| Shah T (27) | Low |
| Shin SH (28) | Low |
| Vaidya R (29) | Moderate |
| Wadhawan R (30) | Low |
| Zozaya C (2021) (31) | Moderate |
| Han SM | Very Low |

## IIb ROBINS-E tool for Risk of Bias

| **First author** | **Before intervention** | | **At intervention** | **After intervention** | | | | **Overall risk of bias** |
| --- | --- | --- | --- | --- | --- | --- | --- | --- |
|  | **Confounding** | **Selection of participants (or into the analysis)** | **Measurement of the exposure** | **Post exposure interventions** | **Missing data** | **Measurement of outcomes** | **Selection of the reported result** |  |
| Berry MJ (16) | High risk (no information about confounding factors or the use of multivariate analysis to correct for these factors) | Low risk | Low risk | High risk | Some concern | Low risk | Low risk | High |
| Blakely ML (17) | Low risk | Some concern (28 cases not enrolled since follow-up was regarded as difficult). | Low risk | Low risk | Low risk | Some concern (no statistical analysis was performed, only absolute values) | Low risk | Some concern |
| Chen S (18) | Some concern | Low risk | Low risk | Low risk | Low risk | Low risk | Low risk | Low |
| Dilli D (19) | Low risk | Low risk | Low risk | Some concern | High risk (12% loss to follow up in NEC group). | Low risk | Low risk | High |
| Fullerton BS (20) | High risk | High risk (not all NEC cases included) | Low risk | High risk | High risk (51% loss to follow up in control group, 45% in NEC group) | Low risk | Low risk | High |
| Han SM (21) | High risk (no information about confounding factors or the use of multivariate analysis to correct for these factors) | High risk | Low risk | High risk | Low risk | Very high risk (large variation in age at follow up) | Some concern | Very high |
| Imren C (22) | Some concern | Low risk | Low risk | Some concern | High risk (55% loss to follow up) | Low risk | Low risk | High |
| Martin CR (23) | Some concern | Low risk | Low risk | Some concern | Low risk | Low risk | Low risk | Some concern |
| Shah T (24) | Low risk | Low risk | Low risk | Some concern | Some concern | Low risk | Low risk | Some concern |
| Shin SH (25) | Low risk | Low risk | Low risk | Some concern | Some concern (50% loss to follow up) | Low risk | Low risk | Some concern |
| Vaidya R (26) | Some concern | Low risk | Low risk | Some concern | Low risk | Low risk | Low risk | Some concern |
| Wadhawan R (27) | Low risk | Low risk | Low risk | Some concern | High risk | Low risk | Low risk | High |
| Zozaya C (2021) (28) | Low risk | Low risk | Low risk | Some concern | Low risk | Low risk | Low risk | Some concern |
